# Effect of Polyphenols on Cognitive Function: Evidence from Population-based Studies and Clinical Trials

**DOI:** 10.1007/s12603-021-1685-4

**Published:** 2021-11-04

**Authors:** Wenzhe Yang, Kaiwang Cui, X. Li, J. Zhao, Z. Zeng, R. Song, Xiuying Qi, Weili Xu

**Affiliations:** 1 Department of Epidemiology and Biostatistics, School of Public Health, Tianjin Medical University, Qixiangtai Road 22, Heping District, 300070, Tianjin, China; 2 Tianjin Key Laboratory of Environment, Nutrition and Public Health, Tianjin, China; 3 Center for International Collaborative Research on Environment, Nutrition and Public Health, Tianjin, China; 4 Department of Respiratory and Critical Care Medicine, The Fifth People's Hospital of Ganzhou, Ganzhou Institute of Respiratory Diseases, Ganzhou, Jiangxi, China; 5 Shandong Provincial Clinical Research Center for Emergency and Critical Care Medicine, Institute of Emergency and Critical Care Medicine of Shandong University, Chest Pain Center, Qilu Hospital of Shandong University, Jinan, Shandong, China; 6 Aging Research Center, Department of Neurobiology, Care Sciences and Society, Karolinska Institutet, Tomtebodavägen 18A Floor 10, SE-171 65 Solna, Stockholm, Sweden

**Keywords:** Polyphenols, nutrition, cognitive decline, dementia, prevention

## Abstract

Due to progressive population aging, a new dementia case occurs at every 3 seconds, placing a heavy burden of disease. Identifying potential risk or preventive factors is emphasized owing to a lack of effective treatment for dementia. There has been emerging evidence on the link of certain dietary components, particularly polyphenols, to brain wellness and cognitive outcomes. Findings from animal and *in vitro* studies appear more consistent and conclusive. However, such an association has not been investigated in depth in human beings. In this review, we examined studies on the effect of dietary polyphenols (including flavonoids, curcumin, and resveratrol) on cognitive function. Intervention in early stages of dementia/Alzheimer's disease might be a target to slow down age-related cognitive decline before disease onset. We summarized 28 epidemiological studies (8 cross-sectional and 20 cohort studies) and 55 trials in this review. Preliminary evidence from epidemiological data provides the necessity for intervention trials, even though the measures of polyphenol intake tend to be less precise. Clinical trials are in favor of the role of some polyphenols in benefiting specific domains of cognition. This review also describes the divergence of results and current limitations of research in this field.

## Introduction

**A**s standards in health care have improved enormously, the lifespan of humanity everywhere is extended, subsequently bringing a higher incidence of aging-related diseases, especially among people over 65 years. Dementia and Alzheimer's disease (AD), as the common neurodegenerative disorders, impact advanced nervous activities in the brain, which in return causes memory decay and disturbances in reasoning, communication, and executive functions (1, 2). Therefore, dementing disorders place a heavy burden on not only patients themselves but their families and society in general, with one new case every 3 seconds worldwide (3). Unfortunately, there is a lack of effective cures for dementia. Therefore, it is critical to maintain or improve brain health to prevent dementia occurrence for general and high-risk population by modifiable factors.

Healthy lifestyle changes, particularly in nutrition patterns, have emerged as a strategy to reduce the burden of aging and age-related diseases. There are a large number of plant-derived components that have broad-ranging beneficial effects on health, and the class of phytochemicals called polyphenols may confer neuroprotective benefits (4, 5). In this context, polyphenol consumption has been proposed to prevent cognitive decline. A growing body of evidence coming from animal and *in vitro* studies has indicated that polyphenols may play an active role in cognitive function (6, 7). However, the effects of polyphenols on human brain have not been investigated in depth. Furthermore, clinical studies on the treatment of polyphenols for dementia or AD, in which there have been already major neuropathology and a substantial loss of neurons in brain preceding so that it is challenging to reverse the damage (8), have revealed unsatisfactory efficacy (9, 10) and thereby the potential role of polyphenols in the prevention of dementia is highlighted. In the current review, we provide an overview of the existing data regarding the potential protective effects of polyphenols on cognitive health, especially in the context of populations at a high risk for dementia, such as the elderly and those with mild cognitive impairment (MCI). We also discuss the limitation of current data and challenges for future research.

## Polyphenols

Polyphenols represent a wide variety of bioactive phytochemicals found in certain foods and beverages, such as fruits, vegetables, herbs, chocolate, coffee, green and black tea, beer, and red wines (11). Based on chemical structures, these compounds are divided into several subclasses: flavonoids, stilbenes, phenolic acids, and lignans (11). Polyphenols have been reported to possess antitumor, anti-inflammatory, antioxidant, anti-thrombotic, and anti-bacteria activities (12, 13), which contribute to various health-related benefits, including being against the progression of cardiovascular disease, diabetes, and cancer (11, 13). Considering that neurodegenerative diseases share certain pathophysiological mechanisms with these disorders, it makes sense that polyphenols have the potential neuroprotective effect (5). The suggested biological mechanisms may involve several typical processes: resistance to amyloidosis, inactivation of free radicals, and inhibition of inflammatory response (14, 15), however these mechanisms have not been fully verified and elucidated. Thus, it is helpful to summarize the research on polyphenols in cognitive disorders to provide evidence for future research.

## Different polyphenols, cognitive decline, and the risk of dementia

### Flavonoids

Flavonoids are the biggest class of phenolics that can be further classified into flavonols, flavones, isoflavones, flavanones, anthocyanidins, and flavanols (catechins and proanthocyanidins) according to the type of their ring structure (11). Apart from total or composite flavonoids, representative dietary sources of specific flavonoids have been concerned broadly (Table 1).

**Table 1:** Summary of select recent human studies that investigated the association of flavonoid intake with cognition

**References**	**Study design**	**Participants**	**Exposures/treatments, duration/follow-up**	**Measures/doses of polyphenol intake**	**Indicators**	**Results**
Cross-sectional studies						
Nurk et al. (2009) (16) Norway	Cross-sectional study	n=2031 (1113 females), 70–74 y	Flavonoid-rich food (including chocolate, wine, and tea) intake	Assessed by comprehensive FFQ	Cognitive function	An association between flavonoid-rich food intake and better cognitive performance
Butchart et al. (2011) (17) UK	Cross-sectional study	n=1091 (543 females), mean age 70 y	Flavonoid (from total fruit intake, total vegetable intake, apples, citrus fruits, tea, chocolate, and red wine) intake	Assessed by SFFQ	Cognitive function	No significant association between flavanone intake and cognitive decline
Igase et al. (2017) (28) Japan	Cross-sectional study	n=152 (91 females), mean age 69.2 y	Equol producer status	Urine samples collected after a soy challenge test	Cognitive function	An association between soy intake and better cognitive performance in equol producers
Hogervorst et al. (2008) (29) Indonesia	Cross-sectional study	n=719 (252 females), 52–98 y	Tofu intake	Assessed by FFQ	Memory function	An association between higher tofu intake and lower memory function
Xu et al. (2015) (30) China	Cross-sectional study	n=517 (284 females), 50–95 y	Tofu intake	Assessed by FFQ	Cognitive function	An association between higher tofu intake and worse cognitive performance
Crichton et al. (2016) (55) USA	Cross-sectional study	n=968 (570 females), 23–98 y	Chocolate intake	Assessed by nutrition and health questionnaire	Cognitive function	An association between more frequent chocolate intake and better cognitive performance
Cohort studies						
Kalmijn et al. (1997) (18) The Netherlands	Cohort study	n=476 (0 females), 69–89 y	Antioxidant intake (including flavonoids), 3 years	Assessed by the cross-check dietary history method adapted to the Dutch situation	Cognitive function	No significant association between flavonoid intake and cognitive decline
Laurin et al. (2003) (19) USA	Cohort study	n=2459 (0 females), 71–93 y	Mid-life flavonoid intake, 8 years	Mean intake of tea (green, black and 11 types of tea infusions)	Dementia and its subtypes	No significant association between flavonoid intake and the risk of dementia or its subtypes
Engelhart et al. (2002) (20) The Netherlands	Cohort study	n=5395 (3183 females), ≥55 y	Flavonoid intake, 6 years	Assessed by SFFQ	Dementia and AD	No significant association between flavonoid intake and the risk of AD
Devore et al. (2010) (21) The Netherlands	Cohort study	n=5395 (3183 females), ≥55 y	Flavonoid intake, 9.6 years	Assessed by SFFQ	Dementia and AD	No significant association between flavonoid intake and the long-term risk of dementia
Commenges et al. (2000) (22) France	Cohort study	n=1367 (801 females), ≥65 y	Flavonoid intake, 5 years	Assessed by nutritional questionnaire	Dementia	An inverse association between flavonoid intake and the risk of dementia
Shishtar et al. (2020) (23) USA	Cohort study	n=2801 (1457 females), ≥50 y	Flavonoid (including flavonols, flavones, flavanones, flavan-3-ols, anthocyanins, flavonoid polymers, and their total intake) intake, 19.7 years	Assessed by SFFQ	ADRD and AD	An association between higher long-term flavonoid intake and lower risks of ADRD and AD
Devore et al. (2012) (24) USA	Cohort study	n=16010 (16010 females), ≥70 y	Flavonoid (including anthocyanidins, flavonols, flavones, flavanones, flavan-3-ols, and polymeric flavonoids) intake, 4 years	Assessed by SFFQ	Cognitive function	An association between higher flavonoid intake and decreased rates of cognitive decline
Letenneur et al. (2007) (25) France	Cohort study	n=1640 (948 females), ≥65 y	Flavonoid (including quercetin, kaempferol, myricetin, luteolin, and apigenin) intake, 10 years	Assessed by FFQ	Cognitive function	An association between higher flavonoid intake and a better cognitive evolution
White et al. (2000) (31) USA	Cohort study	n=3734 (502 females), 71–93 y	Tofu intake	Assessed by frequency of conszumption of 26 specific food and drink items	Brain function and structural changes	An association between higher midlife tofu intake and cognitive impairment and brain atrophy in late life
Calabrò et al. (2019) (56) Italy	Cohort study	n=55 (26 females) with amnestic MCI, 56–75y	Cocoa polyphenol intake, 1 year	240 mg/sachet	Cognitive function	Reduced progression of MCI to dementia
Moreira et al. (2016) (57) Portugal	Cohort study	n=531 (311 females), ≥65 y	Chocolate/caffeine intake, 48 months	Assessed by SFFQ	Cognitive function	An association between chocolate intake/daily caffeine consumption ≤ 75 mg and a lower risk of cognitive decline
Dai et al (2006) (65) USA	Cohort study	n=1589 (863 females), ≥65 y	Fruit and vegetable juices intake, 10 years	Assessed by SFFQ	AD	An association between drinking juices at least 3 times per week and a low risk of AD
Péneau et al. (2011) (66) France	Cohort study	n=2533 (1143 females), 45–60 y	Fruit and vegetables intake, 13 years	Completed 6 records of 24-h dietary record	Cognitive function	An association of fruit and vegetable intake with verbal memory scores, and a negative association with executive functioning scores
Agarwal et al. (2019) (67) USA	Cohort study	n=925 (693 females), 58–98 y	Strawberry intake, 6.7 years	Assessed by FFQ	AD	Associations of strawberries and foods rich in anthocyanidins, and total flavonoids consumption with a decreased risk of AD
Morris et al. (2006) (68) USA	Cohort study	n=3718 (2311 females), ≥65 y	Fruit and vegetable (containing flavonoids) intake, 10 years	Assessed by SFFQ	Cognitive function	No significant association of fruit consumption with slower rate of cognitive decline
Nooyens et al. (2011) (69) The Netherlands	Cohort study	n=2613 (1325 females), 43–70 y	Fruit and vegetable intake, 5 years	Assessed by SFFQ	Cognitive function	No significant association of fruit intake with changes in cognitive function
RCTs						
Kreijkamp-Kaspers et al. (2004) (33) The Netherlands	RCT, DB, PC	n=202 (202 females), 60–75 y	Soy protein, 12 months	25.6 g soy protein (99 mg of isoflavones)	Cognitive function	No significant changes in cognitive function
Fournier et al. (2007) (34) USA	RCT, DB, PC	n=79 (79 females), 48–65 y	Soy isoflavones (soy milk and supplement)/cow's milk and isoflavone supplement, 16 weeks	Soy milk, 72 mg of isoflavones per day/isoflavone supplement, 70 mg of isoflavones per day	Cognitive function	No significant changes in cognitive function
Henderson, et al (2012) (35) USA	RCT, DB, PC	n=350 (350 females), 45–92 y	Isoflavone-rich soy protein 2.5 years	25 g isoflavone-rich soy protein (91 mg aglycone weight of isoflavones) per day	Cognitive function	No significant changes in global cognitive function
Basaria et al. (2009) (36) USA	RCT, DB	n=93 (93 females), mean age 56 y	Soy protein, 12 weeks	20 g (160 mg of total isoflavones) per day	Cognitive function	No significant changes in cognitive function
Ho et al. (2007) (37) China	RCT, DB, PC	n=191 (191 females), 55–76 y	Soy-derived isoflavones, 6 months	80 mg per day	Cognitive function	No significant changes in cognitive function
Santos-Galduróz et al. (2010) (38) Brazil	RCT, DB, PC	n=38 (38 females), 50–65 y	Isoflavones, 4 months	80 mg per day	Cognitive function	Improved performance on information integration
Casini et al. (2006) (39) Italy	RCT, DB, PC, CO	n=78 (78 females), mean age 49.5 y	Phytoestrogens tablets (containing isoflavones), 6 months	600 mg (60 mg of isoflavones) per day	Cognitive function	Improved performance on incidental learning
Kritz-Silverstein et al. (2003) (40) USA	RCT, DB, PC	n=56 (56 females), 55–74 y	Soy-extracted isoflavones, 6 months	110 mg per day	Cognitive function	Improved performance on category fluency
File et al. (2005) (41) UK	RCT, DB, PC	n=50 (50 females), 51–66 y	Isoflavone supplement, 6 weeks	60 mg per day	Cognitive function	Improvements in nonverbal short-term memory and frontal lobe function
Duffy et al. (2003) (42) UK	RCT, DB, PC	n=33 (33 females), 50–65 y	Soy supplement, 12 weeks	60 mg of total isoflavone per day	Cognitive function	Improved performance on recall of pictures and sustained attention task
Thorp et al. (2009) (43) Australia	RCT, DB, CO	n=34 (0 females), 30–80 y	Soy isoflavones, 12 weeks	116 mg per day	Cognitive function	Improvements in spatial working memory, no significant changes in auditory and episodic memory
File et al. (2001) (44) UK	RCT	n=27 (12 females), mean age 25 y	Soy diet, 10 weeks	100 mg of total isoflavones per day	Cognitive function	Improvements in verbal and non-verbal episodic memory, improved performance on letter fluency and planning tests only in females
Gleason et al. (2009) (46) USA	RCT, DB, PC	n=30 (15 females), 62–89 y	Soy isoflavones, 6 months	100 mg per day	Cognitive function	Improvements in visual-spatial memory, construction, verbal fluency, and speeded dexterity
Wightman et al. (2012) (50) UK	RCT, DB, PC, CO	n=27 (16 females), 18–30 y	EGCG, single dose	270 mg, or 135 mg	Cognitive function	No significant changes in cognitive function
Ide et al. (2014) (51) Japan	Pilot study	n=12 (10 females), mean age 88 y	Green tea powder, 3 months	2 g (227 mg of catechins and 42 mg of theanine) per day	Cognitive function	Improved cognitive function
Ide et al. (2016) (52) Japan	RCT, DB, PC	n=33 (29 females), mean age 84.8 y	Green tea powder, 12 months	2 g (220.2 mg of catechins) per day	Cognitive function	No significant changes in cognitive function
Baba et al. (2020) (53) Japan	RCT, DB, PC	n=52 (26 females) with self-assessed cognitive decline, 50–69 y	Catechin capsules, Single dose and 12 weeks	336.4 mg of catechins per day	Cognitive function	Improvements in attention function with a single intake of catechins and memory with a long-term intake
Scholey et al. (2010) (58) UK	RCT, DB, PC, CO	n=30 (17 females), 18–35 y	Cocoa flavanols, single dose	520 mg, 994 mg	Cognitive function	Acute improvements in cognitive function
Field et al. (2011) (59) UK	RCT, single-blinded, CO	n=30 (22 females), 18–25 y	Dark chocolate, single dose	35 g (720 mg of cocoa flavanols)	Cognitive function	Acute improvements in spatial memory and some aspects of the choice reaction time task
Brickman et al. (2014) (60) USA	RCT, DB	n=37 (27 females), 50–69 y	Flavanol intake with or without a regimen of aerobic exercise, 3 months	900 mg of cocoa flavanols and 138 mg of (-)-epicatechin per day	Cognitive function	Improvements in dentate gyrus function
Mastroiacovo et al. (2015) (61) Italy	RCT, DB	n=90 (53 females), mean age 69.55 y	Cocoa flavanols, 8 weeks	993 mg of flavanols per day	Cognitive function	Improved performance on the Trail Making Test A and B, and the Verbal Fluency Test
Desideri et al. (2012) (62) Italy	RCT, DB	n=90 (47 females) with MCI, mean age 71.17 y	Cocoa flavanols, 8 weeks	990 mg of flavanols per day	Cognitive function	Improved performance on the Trail Making Test A and B, and the Verbal Fluency Test
Pase et al. (2013) (63) Australia	RCT, DB, PC	n=78 (54 females), 40–65 y	Cocoa polyphenols, single dose and 30 days	500 mg, 250 mg per day	Cognitive function	No significant changes in cognitive function
Alharbi et al. (2016) (70) UK	RCT, DB, PC, CO	n=24 (0 females), 30–65 y	Flavonoid-rich orange juice, single dose	240 ml (272 mg of flavonoids)	Cognitive function	Acute improvements in cognitive function
Haskell-Ramsay et al. (2017) (71) UK	RCT, DB, PC, CO	n=20 (13 females), 18–35 y	Purple grape juice, single dose	230 ml (32 mg of anthocyanins)	Cognitive function	Acute improvements in aspects of cognition
Lamport et al. (2016) (72) UK	RCT, single-blind, PC, CO	n=24 (20 females), 18–30 y	Citrus juice, single dose	500ml (70.5 mg of flavonoids)	Cognitive function	Acutely improved performance on the Digit Symbol Substitution Test, no improvement in other cognitive tests.
Keane et al. (2016) (73) UK	RCT, DB, PC, CO	n=27 (7 females), 45–60 y	Montmorency tart cherry concentrate, single dose	60 ml (4.08 mg of cyanidin-3-glucoside)	Cognitive function	No significant changes in cognitive function
Miller et al. (2018) (74) USA	RCT, DB, PC	n=37 (24 females), 60–75 y	Freeze-dried blueberry, 90 days	24 g (864 mg of total phenolics and 460.8 mg of anthocyanins) per day	Cognition function	Improvements in executive function
Bowtell et al. (2017) (75) UK	RCT, DB, PC	n=26 (13 females), ≥65 y	Blueberry concentrate supplementation, 12 weeks	30 ml (387 mg of anthocyanidins)	Cognition function	Improvements in working memory and brain activation
Krikorian et al. (2010) (76) USA	Preliminary study	n=9 (4 females) with early memory decline, mean age 76.2y	Wild blueberry juice, 12 weeks	444 ml/d for 54–64 kg, 532 ml/d for 65–76 kg, 621 ml/d for 77–91 kg	Memory function	Improvements in paired associate learning and word list recall
Boespflug et al. (2018) (77) USA	RCT, DB, PC	n=16 (9 females) with MCI, 68–92 y	Blueberry powder, 16 weeks	25g (417 gallic acid equivalents, 269 mg of cyanidin 3-glucoside equivalents) per day	Blood oxygen level-dependent signal (a risk condition for dementia)	Improvements in brain activation
McNamara et al. (2018) (78) USA	RCT, DB, PC	n=76 (41 females) with MCI, 62–80 y	Blueberry powder, 24 weeks	25g (417 gallic acid equivalents, 269 mg of cyanidin 3-glucoside equivalents) per day	Cognitive function	Improvements in memory discrimination
Lamport et al. (2016) (79) UK	RCT, DB, PC, CO	n=25 (25 females), 40–50 y	Concord grape juice, 12 weeks	355 ml (777 mg of total polyphenols) per day	Cognitive function	Improvements in immediate spatial memory
Krikorian et al. (2010) (80) USA	RCT, DB, PC	n=12 (4 females) with MCI, mean age 78.2 y	Concord grape juice, 12 weeks	444 ml/d for 54–64 kg, 532 ml/d for 65–76 kg, 621 ml/d for 77–91 kg	Memory function	Improvements in a verbal learning, no significant changes in verbal and spatial recall
Krikorian et al. (2012) (81) USA	RCT, DB, PC	n=21 (10 females) with MCI, 68–90 y	Concord grape juice, 16 weeks	355 ml/d for 45–57 kg, 444 ml/d for 54–64 kg, 532 ml/d for 65–76 kg, 621 ml/d for 77–91 kg	Memory function and brain activation	Improvements in neurocognitive function
Kean et al. (2015) (82) UK	RCT, DB, CO	n=37 (24 females), 60–81 y	Flavanone-rich 100% orange juice, 8 weeks	500 ml (305 mg of flavanones) per day	Cognitive function	Improvements in global cognitive function
Bookheimer et al. (2013) (83) USA	Preliminary, RCT, DB, PC	n=28 (21 females) with self-reported memory decline, mean age 62.6 y	Pomegranate juice, 4 weeks	8 ounces (contents of polyphenol not reported) per day	Memory tests	Improvements in memory performance and functional brain activation


Abbreviations: FFQ, food frequency questionnaire; SFFQ, semiquantitative food frequency questionnaire; AD, Alzheimer disease; ADRD, Alzheimer disease and related dementias; RCT, randomized controlled trial; DB, double-blind; PC, placebo controlled; CO, cross-over study; EGCG, epigallocatechin gallate; MCI, mild cognitive impairment.

In a couple of cross-sectional studies, Nurk et al. (16) found that intake of flavonoid-rich chocolate, wine, and tea is associated with better cognitive performance in a dose-dependent manner; however, Butchart et al. (17) did not support the role of flavonoids (from total fruit intake, total vegetable intake, apples, citrus fruits, tea, chocolate, and red wine) in the prevention of cognitive decline. Furthermore, previous population-based prospective cohort studies examined the association between dietary intake of antioxidants (including flavonoids) and long-term risk of dementia (18, 19, 20, 21, 22). Contrary to the expectations, most of the results showed that higher intake of foods rich in flavonoids seemed not to modify the risk of dementia or its subtypes. Only Commenges et al. (22) reported an inverse relationship between intake of antioxidant flavonoids and the risk of dementia.

Recently, Shishtar et al. (23) and Devore et al. (24) evaluated the relation between long-term dietary flavonoid intake and cognition in two large cohorts where the intake of flavonoids could be measured repeatedly during the follow-up. The former found that higher dietary intake of total flavonoids (including flavonols, flavones, flavanones, flavan-3-ols, anthocyanins, and flavonoid polymers) is related to a lower risk of AD and Alzheimer's disease and related dementias. The latter one found that greater intake of total flavonoids and berries (high in anthocyanidins) might slow down rates of cognitive decline and delay cognitive aging by as much as 2.5 years in older women. While Letenneut et al. (25) measured the intake of flavonoids only at baseline and showed that higher intake of flavonoids (including quercetin, kaempferol, myricetin, luteolin, and apigenin) is associated with a better cognitive evolution over a 10-year period.

#### Flavonoids in soy and soy-derived products

Isoflavones, one of the better absorbed flavonoids, are specific to soy and soy-based foods, such as soy nuts, tofu, soy milk, and soy butter. In Asian diets, soy isoflavones are more abundant than in Western diets. Isoflavones are also known as phytoestrogens, and their chemical structure is similar to female estrogens. However, it should be noted that there are possible safety issues related to high-dose isoflavone intake, especially from estrogenic and goitrogenic activities (26). Because of such an adverse effect and the lack of consensus regarding the health benefits derived from isoflavone consumption, the American Heart Association does not recommend the use of isoflavone supplements in food or pills (27).

Equol, a metabolite of soy isoflavone, reflects the consumption of isoflavones. Igase et al. (28) conducted a cross-sectional study on the association between equol production status and cognitive function in older adults. In contrast with equol producers, equol non-producers were more susceptible to cognitive decline. Nonetheless, opposite findings deserved attention: two cross-sectional studies in the elderly suggested that higher tofu consumption is associated with lower memory function (29) and worse cognitive performance (30). Furthermore, according to a cohort study, higher tofu consumption in midlife could predict cognitive impairment and brain atrophy in late life among males (31).

Most of the current neuroprotective studies on isoflavones focused on postmenopausal women. These women have lower endogenous estrogen levels after menopause and tend to be at a higher risk of AD (32). A series of randomized controlled trials (RCTs) investigated the effects of soy isoflavones, particularly the estrogen-like actions, on cognition-related aspects. In these studies, doses of total isoflavones varied between 60 and 110 mg/day; periods of intervention ranged from 12 weeks to 2.5 years. Even if almost half of the studies failed to support a significant role of isoflavones supplementation in improving cognitive function (33, 34, 35, 36, 37), others revealed promising results in specific aspects of cognitive function, such as information integration [38], incidental learning (39), category fluency (40), frontal lobe function (41), and recall of pictures and sustained attention task (42).

On the other hand, Thorp et al. (43) paid attention to healthy men. Subjects were randomized to take four capsules (either total containing 116 mg of isoflavone equivalents or placebo) per day for 6 weeks and then they crossed over the alternate treatments for the following 6 weeks. Isoflavone consumption might significantly improve spatial working memory, without being detrimental to the performance on visual-spatial processing. In another RCT by File et al. (44), following 10 weeks of isoflavone administration, young volunteers taking a high soy diet (100 mg/day of total isoflavones) performed better on verbal and non-verbal episodic memory tests, regardless of gender, compared with those taking a low soy diet (0.5 mg/day of total isoflavones). As for tests of letter fluency and planning, the improved performance from the high soy diet group was found only in females. Hogervorst et al. (45) also stated that higher soy intake is related to better immediate recall memory in younger, but not in older people. While Gleason et al. (46) found mixed evidence of the potential cognitive effects of soy isoflavones. In their study design, older adults were randomized to ingest either 100 mg/day of soy isoflavones or placebo for 6 months. When those consumed isoflavones, improvements in the areas of visual-spatial memory, construction, verbal fluency, and speeded dexterity were observed; whereas placebo-treated participants performed better on two tests of executive function.

#### Flavonoids in tea

Tea consumption, with a history of about 5000 years in China, is popular around more than half of the world's population. Tea is derived from the *Camellia sinensis* plant and generally classified into green tea, black tea, and oolong tea according to different fermentation processes (47). With a large quantity of tea polyphenol content and high levels of active enzymes, green tea has been most studied for health benefits. The main components of tea polyphenols are catechins which belong to the flavan-3-ol class of flavonoids, including more than 10 monomers, such as (-)-epigallocatechin-3-gallate (EGCG), (-)-epigallocatechin, (-)-epicatechin-3-gallate, (-)-epicatechin, (-)-catechin-3-gallate, and (-)-gallocatechin-3-gallate, usually among which EGCG dominates.

There has been an increasing body of evidence from observational studies that tea consumption is inversely related to the risk of cognitive disorders in the elderly (48, 49). Wightman et al. (50) conducted a cross-over study in which adults consumed either placebo or two doses (135 mg and 270 mg) of EGCG in counterbalanced order on separate days. Following a 45-min resting absorption period, the intake of EGCG could modulate cerebral blood flow in the frontal cortex but not cognitive performance. Ide et al. assessed the effects of green tea consumption on cognitive function in residents with MCI in their two studies. In a pilot study (51), participants performed better on the Mini-Mental State Examination Japanese (MMSE-J) version than that in baseline when taking 2 g/day green tea powder (containing 227 mg of catechins) over 3 months. Whereas in the RCT (52), after 12-month consumption, changes of MMSE-J score in the green tea powder (containing 220.2 mg of catechins, per day) group and placebo group did not differ significantly. Baba et al. (53) aimed to clarify the role of green tea catechins alone. Subjects with self-assessed cognitive decline took either three catechin capsules (containing 336.4 mg of catechins, caffeine-free) or placebo per day for 12 weeks. An improvement in attention function after a single intake of catechins and beneficial effects of long-term intake on working memory were found.

#### Flavonoids in cocoa

Cocoa, from the dried and fermented seeds of *Theobroma cacao*, originates in the tropical regions of South America. It contains high levels of flavonoids, in particular the flavanols subclass, and is most often consumed in the form of chocolate. The flavanol contents in cocoa products and chocolate differ greatly depending on the cocoa bean variety and origin, agricultural and processing practices, which may be responsible for some mixed outcomes observed in research on the effects of cocoa flavanols on neurocognitive function (54).

A cross-sectional study indicated that consumption of chocolate at least once a week is associated with improved global composite memory, and visuospatial memory and organization (55). Calabrò et al. (56) carried out a retrospective study in which cocoa flavonoids reduced a worsening in cognition assessed by MMSE in patients with MCI. In a prospective cohort study, cocoa/chocolate consumption might slow the cognitive decline in elderly people who consumed less than 75 mg/day of caffeine (57).

Scholey et al. (58) and Field et al. (59) found acute improvements in cognitive performance after consumption of cocoa flavanols. Brickman et al. (60) also revealed that higher cocoa flavanol intake (900 mg/day) for 3 months enhanced dentate gyrus function, considered to be related to age-related memory decline, and thereby improved the cognition in older adults. Mastroiacovo et al. (61) and Desideri et al. (62) designed two similar RCTs in elderly adults and those with MCI, respectively. Subjects were instructed to take a drink containing approximately 990 mg, 520 mg, or 45 mg of cocoa flavanols once daily over 8 weeks. The performance on the Trail Making Test A and B as well as the verbal fluency test was significantly better in those with the highest flavanol intake, for whichever population. Pase et al. (63) examined the acute and sub-chronic neuroprotective effects of cocoa polyphenols. Participants were individually assigned to receive a daily dark chocolate drink mix containing 500 mg, 250 mg of polyphenols, or placebo for 30 days. However, cognitive aspects were unchanged by any dose of administration at all-time points.

#### Flavonoids in fruits

Fruits and fruit juices are excellent sources of flavonoids. Anthocyanins are mainly found in red and blue fruits, in particular berries, such as blueberries, raspberries, red grapes, and cherries (13). Flavanones are present in high concentrations in citrus fruit, involving naringenin from grapefruit, hesperetin from oranges, and eriodictyol from lemons (64). Additionally, 100% fruit juice consumption is recommended compared with nutrient-poor sugar-sweetened beverages (5).

Epidemiological data suggested a link between fruit (juice) consumption and age-related cognitive decline. In a cohort study of older dementia-free Japanese Americans, the consumption of fruit/vegetable juice, containing a high concentration of polyphenols, is inversely related to AD risk. And the effect was independent of vitamin C, E, β-carotene, and tea consumption, indicating that polyphenols in fruit/vegetable juice might be responsible (65). Péneau et al. (66) observed the beneficial effect of consumption of fruits and vegetables or fruits alone on the performance on verbal memory after a 13-year follow-up. Agarwal et al. (67) found that higher intake of strawberry rich in anthocyanidins is associated with a decreased risk of AD during the mean 6.7 years of follow-up. Nevertheless, Morris et al. [68] and Nooyens et al. (69) reported that higher vegetable but not fruit consumption might be associated with less cognitive decline in the cohorts.

Some RCTs addressed the acute effects of polyphenols from fruits. Alharbi et al. (70) stated that middle-aged males taking a 240 ml of orange juice (containing a total of 272 mg of flavonoids) performed better significantly on the tests of objective and subjective cognition over the course of 6 hours. Haskell-Ramsay et al. (71) found that 230 ml of grape juice (consisting of Welch's™ purple grape juice plus Schweppes™ blackcurrant favour cordial, containing 32 mg of anthocyanins) intake enhanced aspects of cognition in young adults after a 20-min absorption period. Lamport et al. (72) observed a role of 500 ml of citrus juice (containing 70.5 mg of flavonoids) intake in increasing cerebral blood flow and improving the performance in the digit symbol substitution test but no other behavioral cognitive tests among young adults 2 hours following the consumption. Likewise, Keane et al. (73) showed that cherry concentrate consumption could modulate some variables of vascular function immediately rather than cognition.

Clinical evidence reporting similar chronic benefits also existed. Numerous investigations showed that blueberry products appeared to contribute to brain health in older adults, such as executive function (74), working memory and brain activation (75), paired associate learning and word recall (76), brain activation (77), and memory discrimination (78). Concord grape juice (by Welch Foods, Inc) supplementation was reported to improve spatial memory and driving performance in middle-aged working mothers (79) and memory function in older adults with MCI (80, 81). Kean et al. (82) found that 8-week, daily 500 ml of 100% orange juice (containing 305 mg of flavanones) consumption was beneficial for global cognitive function. A daily 8-ounce pomegranate juice (the commercial Pom Wonderful product, contents of polyphenol not reported) consumption for 4 weeks was also found to benefit memory performance, perhaps affected through increased task-specific cerebral blood flow, and functional brain activation among older persons with self-reported memory decline (83). Furthermore, Nilsson et al. (84) focused on a mixed berry juice that consisted of blueberries, blackcurrant, elderberry, lingonberries, strawberry, and tomatoes, with 795 mg daily amounts of total polyphenols. Subjects performed better on the working memory test after the 5 weeks of intervention.

### Curcumin

Curcumin is a yellowish pigment and a well-known nonflavonoid polyphenol, belonging to the group of phenolic acids. Curcumin is the major bioactive component in *Curcuma longa* that has a long history of application as a dye, a spice, and as an anti-inflammatory agent in traditional medicines in China and India (85). Notably, because curcumin itself displays low solubility in water and a poor pharmacokinetic profile, novel curcumin formulations that make up for these defects and are available commercially are in common use in human trials (Table 2).

**Table 2:** Summary of RCTs that investigated the association of curcumin intake with cognition

**References**	**Study design**	**Participants**	**Exposure/treatment, duration/follow-up**	**Dose of polyphenol intake**	**Indicators**	**Results**
DiSilvestro et al. (2012) (86) USA	RCT, PC	n=38 (34 females), 40–60 y	Lapidated curcumin, 4 weeks	400 mg Longvida® Optimized Curcumin (80 mg of curcumin) per day	Health promoting effects	A decrease of plasma amyloid β-protein concentrations
Cox et al. (2015) (87) Australia	RCT, DB, PC	n=60 (38 females), 60–85 y	Curcumin formulation, single dose and 4 weeks	400 mg Longvida® Optimized Curcumin (80 mg of curcumin)	Cognitive function	Improved performance on sustained attention and working memory tasks after 1 hour and working memory following 4 weeks
Lee et al. (2014) (88)) Chinese Taiwan	RCT, DB, PC	n=48 (25 females), ≥60 y	Turmeric, single dose	1g (contents of curcumin not reported)	Working memory	Improvements in working memory
Small et al. (2018) (89) USA	RCT, DB, PC	n=40 (22 females), 51–84 y	Curcumin formulation, 18 months	180 mg of curcumin per day	Cognitive function	Improvements in memory and attention performance
Rainey-Smith et al. (2016) (90) Australia	RCT, DB, PC	n=96 (68 females), 40–90 y	Curcumin formulation, 12 months	1500 mg of Biocurcumax™ (88% curcuminoids) per day	Cognitive function	No significant changes in cognitive function


Abbreviations: RCT, randomized controlled trial; DB, double-blind; PC, placebo controlled.

DiSilvestro et al. (86) investigated various actions of a low dose of curcumin on wellness-related measures. This study showed a decrease of plasma amyloid β-protein concentrations after a daily supplement of lipidated curcumin (Longvida® Optimized Curcumin from *Curcuma Longa* root, containing 80 mg of curcumin) for 4 weeks, which was associated with the mechanism by which curcumin might influence brain aging and the development of AD. Moreover, with prolonging the duration of intervention, this decrease could become larger, suggesting curcumin might produce potential health promotion on cognition. An RCT by Cox et al. (87) was the first study to explore the roles of curcumin on cognition and reported significant acute (one hour after a single dose) effect of solid lipid curcumin formulation (Longvida® Optimized Curcumin, containing approximately 80 mg of curcumin) on enhanced performance on sustained attention and working memory tasks. After a chronic daily treatment (4 weeks), working memory and mood were also significantly improved. This study provided clues that curcumin even at a low dose had the potential to improve cognitive function. Lee et al. (88) focused on a role of turmeric on post-prandial working memory in elderly adults with pre-diabetes but dementia-free. It showed that ingestion of turmeric (1 g, contents of curcumin not reported) increased working memory independent of amyloid precursor protein, the biomarker of AD, 6 hours after supplementation. A recent long-term (18 months) RCT by Small et al. (89) displayed that taking Theracurmin® twice daily (i.e., 180 mg/day of curcumin) might make for significant memory and attention benefits, which was associated with decreases in amyloid and tau accumulation in brain regions. Rainey-Smith et al. (90) carried out a 12-month RCT where community-dwelling adults with good health received either 1500 mg/day Biocurcumax™ (containing 88% curcuminoids and 7% volatile oil) or placebo. No significant difference in cognitive performance after supplementation was observed except for a significant time × treatment group interaction on the Montreal Cognitive Assessment (MoCA). Nevertheless, this association was owing to a decline in MoCA scores of the placebo group at 6 months that was not observed in the curcumin treatment group. And at 12 months, there was also nonsignificant difference in the MoCA scores between the two groups.

### Resveratrol

Resveratrol is one of the most intensively studied among the stilbene compounds with mother-nucleus of distyrenes. It was first isolated from white hellebore (*Veratrum grandiflorum* O. Loes) roots in 1940 and later isolated from *Polygonum cuspidatum* roots in 1963. To date, this natural polyphenol resveratrol has been detected in more than 70 plant species (91). However, dietary sources of resveratrol are relatively limited and are mainly red wines, peanuts, pistachios, berries, and grapes (92). The compound is determined in two isomers: *cis*-isomer and *trans*-isomer, of which *trans*-resveratrol is concerned by most research due to its greater chemical stability and wider biological activities (93).

Both Kennedy et al. (94) and Wightman et al. (95) focused on the acute effect of resveratrol with regard to cerebral blood flow and cognitive performance in young adults. The former found that subjects' cerebral blood flow increased after a 45-min resting absorption of resveratrol (250 mg or 500 mg), but with little change in cognitive function. Given the hypothesis that piperine could alter resveratrol pharmacokinetics and enhance its bioavailability (96), the latter further ascertained the combined effect of resveratrol and piperine. Similar to the results above, only cerebral blood flow instead of cognitive performance was improved after a 40-min rest/absorption period, despite with a co-supplementation of 250 mg of resveratrol with 20 mg of piperine. In addition, the findings indicated that piperine seemed not to induce an overall increase in the bioavailability of resveratrol. Wong et al. (97) also studied the acute (75 min after a single dose) effect of different resveratrol concentrations in patients with type 2 diabetes but found no significant change in overall cognitive performance. While patients performed better on the multitasking test battery that assessed visual scanning and attention, working memory, and executive function, which might be correlated with the enhanced neurovascular coupling capacity, after taking 75 mg or 300 mg of resveratrol.

On the other hand, Wightman et al. (98) conducted an RCT to explore the chronic effect of 28-day supplementation of 500 mg of resveratrol. But it was little beneficial for aspects of cognitive function except accuracy of the 3-Back task in young people, even if on day 1 there was the effect on more accurate but slower serial subtraction task performance. Moran et al. (99) investigated the impact of a daily multi-ingredient supplementation (containing 150 mg of resveratrol) for 6 months. But a limited beneficial impact on overall cognitive function and composite domains (including executive function, memory, attention, sensorimotor speed, motor imagery accuracy, and subjective awareness of cognitive failures) was captured, either. Whereas Anton et al. (100) reported that compared with 300 mg/day of resveratrol and placebo, 1000 mg/day of supplementary resveratrol for 90 days improved psychomotor speed but in the absence of other domains of cognitive function in older overweight adults. Moreover, Witte et al. (101) and Huhn et al. (102) assessed the role of resveratrol in the elderly who were overweight and who were with a wide body-mass index range, respectively. In both two studies, subjects were randomized into either 200 mg/day of resveratrol or placebo for 26 weeks. Contradictory results were obtained: the first study found significant effects of resveratrol on retention of memory and an increase in functional connectivity of hippocampus with frontal, parietal, and occipital areas, which is a key region involved in memory function. Furthermore, resveratrol could benefit glucose metabolism that might further mediate neuronal function and cognitive performance. The second study failed to find any improvement in verbal memory and changes in hippocampus volume, microstructure, and functional connectivity. Furthermore, another 26-week trial investigated whether the beneficial impacts of resveratrol could extend to patients with MCI (103), in which resveratrol supplementation at a dose of 200 mg/day could preserve hippocampus volume and improve hippocampal resting-state functional connectivity. Whereas no significant influence of resveratrol on memory performance or microstructure of hippocampal neurogenesis was detected. In post-menopausal women, 75 mg of resveratrol ingested twice daily over 14 weeks resulted in better cognitive performance, which might be at least partially driven through improvement in cerebrovascular responsiveness to cognitive stimuli (104). Also, in such a population, other researchers hypothesized that by ameliorating circulatory function and regional blood flow in the brain, resveratrol could enhance mood and cognition (105) (Table 3).

**Table 3:** Summary of RCTs addressing the association of resveratrol intake with cognition

**References**	**Study design**	**Participants**	**Exposure/treatment, duration/follow-up**	**Dose of polyphenol intake**	**Indicators**	**Results**
Kennedy et al. (2010) (94) UK	RCT, DB, PC, CO	n=22 (18 females), 18–25 y	Resveratrol, single dose	250 and 500 mg	Cognitive function	No significant changes in cognitive function
Wightman et al. (2014) (95) UK	RCT, DB, PC, CO	n=23 (19 females), 19–34 y	Resveratrol with or without piperine, single dose	250 mg (with or without 20 mg of piperine)	Cognitive function	No significant changes in cognitive function
Wong et al. (2016) (97) Australia	RCT, DB, PC	n=36 (10 females), 40–80 y	Resveratrol, single dose	75, 150 and 300 mg	Cognitive function	Improved performance on the multi-tasking test battery with 75 mg and 300 mg resveratrol
Wightman et al. (2015 (98) UK	RCT, DB, PC	n=60 (51 females), 18–30 y	Resveratrol, 28 days	500 mg per day	Cognitive function	Improved perfor mance on accuracy of the 3-Back task but not other aspects of cognitive function
Moran et al. (2018) (99) Ireland	RCT, DB, PC	n=37 (19 females), 68–83 y	Liquid juice, 6 months	200 ml (150 mg of resveratrol)	Cognitive function	No significant changes in overall cognitive function or composite cognitive domains
Anton et al. (2018) (100) USA	RCT (Phase IIa), DB, PC	n=32 (16 females), 65–93 y	Resveratrol, 90 days	High-dose (1000 mg/d)	Cognitive function	Improvements in psychomotor speed, no significant changes in other domains of cognitive function
Witte et al. (2014) (101) Germany	RCT, DB, PC	n=46 (18 females), 50–75 y	Resveratrol, 26 weeks	200 mg per day	Memory performance	Improvements in memory performance and functional connectivity of hippocampus
Huhn et al. (2018) (102) Germany	RCT, DB, PC	n=53 (28 females), 60–79 y	Resveratrol, 26 weeks	200 mg per day	Verbal memory performance	No significant changes in verbal memory performance
Köbe et al. (2017) (103) Germany	RCT, DB, PC	n=40 (21 females) with MCI, 50–80 y	Resveratrol, 26 weeks	200 mg per day	Memory performance	Improvements in functional connectivity of hippocampus, no significant changes in memory performance
Evans et al. (2017) (104) Australia	RCT, DB, PC	n=80 (80 females), 45–85 y	Resveratrol, 14 weeks	150 mg per day	Cognitive function	Improved performance on cognitive tasks in verbal memory and in overall cognitive performance
Evans et al. (2016) (105) Australia	RCT, DB, PC	n=80 (80 females), 45–85 y	Resveratrol, 14 weeks	150 mg per day	Cognitive function	Improvements in cognitive function


Abbreviations: RCT, randomized controlled trial; DB, double-blind; PC, placebo controlled; CO, cross-over study; MCI, mild cognitive impairment.

### Other polyphenols

Kreijkamp-Kaspers et al. (106) performed a cross-sectional study to explore the possible influence of isoflavone and lignan intake on cognitive function in women. As was reported, high lignan intake was associated with better processing capacity RCT, DB, PC and speed, and executive function. Nevertheless, no significant association between taking isoflavones and cognitive function was observed. These findings were replicated in another cross-sectional study in postmenopausal women in the same locality (107). Likewise, Nooyens et al. (108) follow antioxidants (including flavonoids and lignans) with interest and found that higher lignan intake is associated with less decline in global cognitive function, memory, and speed processing while higher flavonoid intake is also related to cognitive flexibility.

Rabassa et al. (109) carried out a cohort study with 3 years of follow-up in an older population. High concentration of total urinary polyphenols, a nutritional biomarker of dietary polyphenols, was reported to be associated with a lower risk of cognitive decline. However, no significant effect of total dietary polyphenols on cognition was observed. Another large prospective study with a 13-year follow-up identified the associations of total or class-specific polyphenol intake with cognitive function (110). According to this study, there was a significant association of higher total polyphenol intake with better performance on verbal memory rather than executive function. More specifically, intake of total flavonoids, catechins, theaflavins, flavonols, and hydroxybenzoic acids was positively associated with language and verbal memory. Unexpectedly, some specific polyphenols, such as catechins, proanthocyanidins, and flavonols, might have an adverse effect on executive function. Lefèvre-Arbogast et al. (111) examined the long-term (12 years) association between the intake of 26 polyphenol subclasses and dementia risk in a 3-city cohort. This study identified that a pattern of polyphenol intake combining several flavonoids (including dihydroflavonols, anthocyanins, isoflavonoids, and flavanones), stilbenes (including resveratrol), lignans, and other subclasses is associated with a lower risk of all-cause dementia and AD. Furthermore, Lee et al. (112) concerned for patients with mild decline in cognition who were at a high risk for dementia. Participants were randomized to take either 72 g/day active grape formulation (in which flavonoids and resveratrol abundantly exist) or placebo over 6 months. There were declines in region of the brain having metabolism in relation to aging and development of early stages of AD in the placebo group, yet grape consumption could protect against those declines, which in turn contribute to the improvement in attention and working memory performance (Table 4).

**Table 4:** Summary of select recent human studies that investigated the association of other polyphenol intake with cognition

**References**	**Study design**	**Participants**	**Exposure/treatment, duration/follow-up**	**Measures/dose of polyphenol intake**	**Indicators**	**Results**
Kreijkamp-Kaspers et al. (2007) (106) The Netherlands	Cross-sectional study	n=301 (301 females), 60–75 y	Isoflavone and lignan intake	Assessed by FFQ	Cognitive function	An association of higher lignan intake with better processing capacity and speed, and executive function, no association between isoflavone intake and cognitive function
Franco et al. (2005) (107) The Netherlands	Cross-sectional study	n=394 (394 females), mean age 66.3 y	Isoflavone and lignan intake	Assessed by FFQ	Cognitive function	An association between higher lignan instead of isoflavone intake with better cognitive performance
Nooyens et al. (2015) (108) The Netherlands	Cohort study	n=2613 (1325 females), 43–77 y	Antioxidant (including flavonoids and lignans) intake, 5 years	Assessed by SFFQ	Cognitive function	An association of higher lignan intake with less decline in global cognitive function, memory, and speed processing, an association of higher flavonoid intake with cognitive flexibility
Rabassa et al. (2015) (109) Italy	Cohort study	n=652 (361 females), ≥65 y	Polyphenol intake, 3 years	Assessed by FFQ	Cognitive function	No significant changes in cognitive function
Kesse-Guyot et al. (2012) (110) France	Cohort study	n=2574 (1161 females), mean age 66 y	Total or class-specific polyphenol intake, 13 years	Assessed by 24-h dietary record	Cognitive function	An association of high total polyphenol intake with verbal memory but not with executive function, an association of total flavonoid, catechin, theaflavin, flavonol, and hydroxybenzoic acid intake with language and verbal memory
Lefèvre-Arbogast et al. (2018) (111) France	Cohort study	n=1329 (825 females), mean age 75.8 y	Polyphenol subclass intake, 11.7 years	Assessed by 24-hour dietary recall	Dementia and AD	An association between a specific pattern of polyphenol intake and a lower risk of all-cause dementia and AD
Lee et al. (2017) (112) USA	Pilot study	n=10 (5 females) with MCI, 66–82 y	Active grape formulation (including flavonoids and resveratrol), 6 months	72 g (371.25 mg of total polyphenols) per day	Cognitive function	Decreased cognitive decline


Abbreviations: SFFQ, semiquantitative food frequency questionnaire; FFQ, food frequency questionnaire; AD, Alzheimer disease; MCI, mild cognitive impairment.

## Discussion

Our search retrieved published 28 epidemiological studies and 55 clinical trials exploring the roles of polyphenols in cognitive outcomes. Polyphenol intake appears safe and well-tolerated with no excess side effects within the ranges of dose used and the duration of studies. Overall, there has been promising evidence that polyphenol-rich foods including soy, tea, cocoa, fruits, and related products, as well as curcumin and resveratrol, have the potential to improve cognition and increase resistance to age-related cognitive decline, at least among individuals without dementia or AD.

Despite the findings from some studies where the observed indicators are the incidence of dementia/AD or gross cognition are conflicting, the more inspiring results come from studies that have applied multiple measures on the specific cognitive domains. Acute benefits of certain polyphenols after a single dose are likely, in particular on attention function and working memory; longer-term intake is necessary to achieve observable effects. These polyphenols may have the most notable effect on memory and executive function. This is in accordance with possible mechanisms underlying the neuroprotective effects of polyphenols, namely by improvement of cerebral blood flow and connectivity of hippocampus as well as reduction of oxidative stress and neuroinflammation function (113, 114), which are specifically related to the cognitive process in memory and frontal executive function (115, 116). In addition, these benefits may be also relevant for younger adults. The ongoing study on polyphenol consumption in older adults at risk for dementia/AD is significant, while it is possible that the window of opportunity to ameliorate the age-related cognitive deterioration by nutritious diet emerges early in the life course (117).

In fact, the bioavailability of oral natural polyphenols is low in humans, among which the resveratrol bioavailability is even less than 1%, regardless of dose escalation and repeated dose administration (118). The states of polyphenols and matrix in which polyphenols present influence their bioavailability. Thus, it is plausible that the combination of polyphenols with other diets may exert synergistic effects on cognitive wellness. For instance, the bioavailability of tea EGCG is increased when co-administrated with caffeine (119). After an administration accompanied by piperine, the bioavailability of curcumin or resveratrol is strongly elevated (96). One previous literature has also suggested that combing specific polyphenols could be more relevant for brain fitness than a single supplement of polyphenol subclasses (111). Thus, identification of synergism between polyphenols and other nutritions becomes an area of interest. On the other hand, diverse formulations have been produced to enhance the oral bioavailability of polyphenols. In the formulation Longevinex®, resveratrol is supplemented with 5% quercetin and 5% rice bran phytate and these ingredients are micronized to increase the bioavailability (8). Curcumin formulations (Longvida® Optimized Curcumin, Biocurcumax™, and Thercurcumin®) have been also shown to yield 27- to 185-fold bioavailability compared with conventional curcumin (120). When dietary supplementation of polyphenols is regarded as a novel therapeutic approach, its poor bioavailability needs to be overcome.

The studies in this review are concentrated in non-dementia human subjects, resulting in a modest number of articles and inconsistent results exist. Methodological aspects must be considered carefully. With regard to the majority of observational studies, assessment of polyphenol intake levels tends to be not accurate enough as a result of under- or overestimation of food consumption from self-reports and differences in the assessment of nutrient composition with inconsistent food composition tables. As an example, a single 24-hour dietary recall may not represent usual food selection (19). Moreover, the measurements of diet at one point cannot reflect long-term intake owing to possible changes in dietary practices over the years. There remain discrepancies regarding the cognitive performance measured and the tasks used, and there is even quite a little confusion on the terms applied for the same test, which makes across-study comparisons difficult. Besides, some tests of global cognition used to screen cognitive deficits or dementia, such as the MMSE, appear not sensitive enough to detect subtle domains. It could be more powerful to utilize demanding neuropsychological tests covering a wider range of functions. If conditions permit, the use of functional magnetic resonance imaging can better support findings from cognitive screenings. On the other hand, the durations of intervention range from 4 weeks to 2.5 years in non-acute RCTs, but with no obvious association between intervention duration and its efficacy, and it remains unclear how long it will take to induce significant changes in cognition after a related supplementation. In addition, many factors to which cognitive function is sensitive should be taken into account, such as mood, sleep, physical activities, other illnesses, and genetic factors (5, 117). Finally, RCTs can provide stronger evidence on the causal association of polyphenol intake with cognitive function; nevertheless, the sample sizes are relatively small, with fewer than 100 participants in the majority of studies, and the effect of short-term supplementation in clinical studies may not be comparable with long-term dietary intake (20). Therefore, future investigations with a larger sample of subjects (particularly in pre-clinical or MCI stage), longer intervention periods, and longer follow-up after the end of supplement use should determine whether those benefits can be validated and transferred to humans.

## Conclusion

In the absence of curative treatment, nutrients from health diet are important modifiable factors for cognitive aging and dementia. Polyphenols, particularly flavonoids, curcumin, and resveratrol, have pleiotropic biological activities capable of being ideal candidates to preserve or improve cognitive performance that precedes the onset of dementia/AD. Certainly, discrepancies in study design partly bring about inconsistent findings on the same component. Noteworthy, it is essential to identify the effective dose and duration of supplementation, enhance the bioavailability of substances, and establish standardized preparation, to ensure polyphenol levels sufficient for the protective action. Furthermore, the possibility of interactions with other bioactives present in foods or that can be administered as supplements (e.g., fiber, terpenoids, and alkaloids) deserves consideration. Even if human polyphenol research on cognitive function is at an early stage and much work needs to be done, the observed associations are promising and call for future investigation.
